# The complete mitochondrial genome of *Hemiboea subcapitata* (Gesneriaceae) and comparative analysis across Lamiales

**DOI:** 10.3389/fpls.2026.1921030

**Published:** 2026-08-12

**Authors:** Ru-Yang Ye, Peng Zhou, Xiao-Guo Xiang, Jin-Long Liu

**Affiliations:** 1 School of Life Sciences, Institute of Advanced Agricultural Sciences, Nanchang, Jiangxi, China; 2 School of Life Sciences, Key Laboratory of Poyang Lake Environment and Resource Utilization Ministry of Education, Nanchang University, Nanchang, Jiangxi, China

**Keywords:** *Hemiboea subcapitata*, Lamiales, mitochondrial genome, phylogeny, selective pressure

## Abstract

**Introduction:**

The mitochondrial genomes of plants in extreme environments often harbor distinctive evolutionary signals. However, the differences between karstic and non-karstic species remain unclear.

**Methods:**

In this study, the complete mitochondrial genome of karstic species *Hemiboea subcapitata* (Gesneriaceae) was assembled and annotated.The selective pressure, nucleotide diversity and nucleotide substitution spectrum within Lamiales were conducted to investigate the adaptation pattern of mitochondrial protein-coding genes (PCGs) between karstic and non-karstic species. Further, the phylogenetic conflicts of Lamiales based on mitochondrial and chloroplast PCGs were assessed.

**Results:**

The assembled mitochondrial genome of *H. subcapitata* is a circular molecule of 621,547 bp with 43.6% GC content, and 67 genes were annotated, including 37 PCGs, 27 tRNA genes, and 3 rRNA genes. Our analyses identified 121 SSRs and 450 RNA editing sites of *H. subcapitata*. Within Gesneriaceae, the mitochondrial showed the diverse genome structure and gene composition, and the conserved GC content. Strikingly, three genes (*atp4*, *atp8*, and *ccmB*) exhibited elevated nonsynonymous/synonymous ratios (Ka/Ks) both in karstic and non-karstic Lamiales species, while only rps10 with Ka/Ks >1 in karstic species. The mutational spectrum showed that transition was the predominant type in both karstic and non-karstic Lamiales species. Furthermore, five genes (*atp1, atp9, rpl2, rpl5* and *sdh3*) with highest Pi values were the candidate hypervariable regions of Lamiales. Phylogenetic conflicts between mitochondrial and chloroplast PCGs was mainly related to incomplete lineage sorting.

**Discussion:**

This study revealed the differences of mitochondrial genomes in karstic and non-karstic species plants across Lamiales and highlighted the impact of habitat on organellar genome evolution.

## 1 Introduction

Mitochondria are central to cellular respiration and energy conversion, playing indispensable roles in plant growth, development, and stress responses (Millar et al., 2011; Liberatore et al., 2016). Recent studies have considered mitochondria as important environmental sensors, capable of perceiving stressful conditions and triggering gene expression and epigenomic changes in plants (Dopp et al., 2021). Accumulating evidences have suggested that the plant mitochondrial genomes from extreme environments often display special evolutionary signals. For instance, comparative analysis of the mitochondrial genome of *Geoffroea decorticans*, a legume tree native to the Atacama Desert, provided evidence for adaptive evolution under extreme drought. This adaptive signature is reflected in the highest proportion of plastid-derived DNA reported among angiosperms (Contreras-Díaz et al., 2023). Similarly, *Hippophae tibetana* (Elaeagnaceae) in high-altitude habitat exhibited significant signals of positive selection in multiple mitochondrial genes, including *atp6*, *ccmB*, *nad4L*, and *nad7*, which might serve as an adaptive strategy for extreme environments (Zeng et al., 2024). Therefore, it is essential to explore the adaptive features of mitochondrial genomes under different stress.

Karst environments were characterized by low soil water content, periodic water deficiency and poor nutrient availability (Li et al., 2021a; Jiang et al., 2025). Yet, there are about 226 families, 8795 species in karsts, *ca.* 10% of which belong to Lamiales (Yu et al., 2017). In recent years, a few studies reported the mitochondrial genomes of Lamiales plants inhabited in karst environments. Mitochondria of these species generally exhibit diverse genome structures and frequent chloroplast DNA transfers (Zhang et al., 2012; Zhong et al., 2022). For instance, the mitochondrial genome of the karst-endemic plant *Dorcoceras hygrometrica* (Gesneriaceae) consisted of a master circle and four isomeric molecules, which contained 10.5% chloroplast-derived sequences (Zhang et al., 2012). Zhong et al. (2022) assembled the mitogenome of *Aeginetia indica* (Orobanchaceae), a holoparasitic species often distributed in karsts, and showed that it forms a main circular molecule that generates multiple subgenomic circles via active repeat-mediated recombination. Compared to the karstic species, the mitogenome of the non-karstic species *Lavandula angustifolia* (Lamiaceae) was a typical circular structure with 2.94% chloroplast-derived sequences (Wang et al., 2024). A survey of 11 non-karst Lamiaceae species revealed that four mitochondrial genes (*ccmFC, nad7, nad4*, and *ccmFN*) showed positive selection (Chetri et al., 2025). Due to the different characters between the mitochondrial genomes of karstic and non-karstic Lamiales species, comparative analyses of these genomes would be significant to reveal the adaptations of plants in karsts.

Previous studies have reconstructed the phylogenetic relationships of Lamiales using chloroplast and mitochondrial genes, but the results from these different molecular markers remain inconsistent for some families (Li et al., 2021b; Tao et al., 2026). Specifically, based on 80 chloroplast genes, Li et al. (2021b) found that Verbenaceae was sister to Bignoniaceae, while Tao et al. (2026) employed 25 conserved mitochondrial protein-coding genes (PCGs) and showed that Verbenaceae was sister to Lentibulariaceae. Hybridization and incomplete lineage sorting (ILS) have been proposed to be the primary causes of phylogenetic discordances between chloroplast and mitochondrial genomes (e.g., Xue et al., 2024; Liang et al., 2025). However, the underlying causes of phylogenetic conflicts between the two organellar genomes within Lamiales remain unclear.

Here, we focused on *Hemiboea subcapitata*, a perennial herbaceous plant in Gesneriaceae (Lamiales) to investigate the potential mitochondrial adaptations in karsts. This species mainly inhabits in karst regions of Southern China, is also a commonly used folk medicinal plant in the Guangxi Zhuang Autonomous Region, mainly for the treatment of sore throat, measles, and burns (Li and Wang, 2005). Wang et al. (2025) published the chromosome-level genome of *H. subcapitata* and identified 25 key genes in the flavonoid pathway and candidate MYB transcription factors related to environmental adaptation in karsts. The high quality HiFi data of *H. subcapitata* provided a solid data foundation for successfully assembling its mitochondrial genome characteristics and subsequent analyses of mitochondrial adaptations in karsts.

In this study, we newly assembled and annotated the complete mitochondrial genome of *H. subcapitata*. Then we characterized four key features of the mitochondrial genome of this species, including codon usage bias, RNA editing sites, repetitive sequences, and horizontal sequence transfer. To explore the adaptative pattern of mitochondrial PCGs in karstic and non-karstic Lamiales species, we examined the mutational spectrum, evaluated selective pressures and nucleotide diversity. Furthermore, we reconstructed the phylogenetic trees across 49 Lamiales species using mitochondrial and chloroplast genomes to investigate the primary reason of topological conflicts. Collectively, our study aims to compare the adaptive divergence of mitochondrial PCGs between karstic and non-karstic species, and to assess the phylogenetic consistency of organellar genomes in Lamiales.

## 2 Materials and methods

### 2.1 Mitochondrial genome assembly and annotation

The mitochondrial genome of *H. subcapitata* was *de novo* assembled using PacBio circular consensus sequencing (CCS) HiFi reads from Wang et al. (2025). The assembly was performed with OATK v1.0 (Zhou et al., 2025) under default parameters to ensure the continuity and accuracy of sequence scaffolding. Following initial assembly, the generated assembly graph was visualized using Bandage v0.7.1 (Wick et al., 2015). To validate the assembly and assess sequencing depth, the genomic clean reads were mapped back to the assembled mitochondrial genome using BWA v0.7.17 (Li and Durbin, 2009), and sequencing depth was calculated with SAMtools v1.8 (Danecek et al., 2021).

For mitochondrial genome annotation, the Plant Mitochondrial Genome Annotator (PMGA; Li et al., 2024) was used for preliminary annotation to rapidly identify functional elements, including PCGs, ribosomal RNAs (rRNAs), and transfer RNAs (tRNAs). In addition, the annotation was manually adjusted in Geneious v2025.0.2 (Kearse et al., 2012) with *Hemiboea yongfuensis* (GenBank accession number: PX394567) as a reference. The positions of start and stop codons of all PCGs were verified to correct annotation errors and ambiguous gene structure boundaries. Finally, the OGDRAW (https://chlorobox.mpimp-golm.mpg.de/OGDraw.html) was employed to visualize the annotation results of the mitochondrial genome.

### 2.2 Comparative analyses of mitochondrial genome

The 37 PCGs of the mitochondrial genome were obtained via Phylosuite v1.2.2 (Zhang et al., 2020a). The codon usage of PCGs was analyzed by calculating relative synonymous codon usage (RSCU) values using Mega v11.0 (Tamura et al., 2021). An RSCU value above 1 represents strong codon usage bias, a value of 1 indicates neutral codon usage, and a value below 1 corresponds to weak codon preference (Liu et al., 2020).

Simple sequence repeats (SSRs) were detected using MISA (Beier et al., 2017; http://pgrc.ipkgatersleben.de/misa/) with repeat unit sizes ranging from 1 to 6 nucleotides. The minimum repeat number thresholds were set to 10, 5, 4, 3, 3, and 3 for 1- to 6-nucleotide repeats, respectively. Tandem repeats were identified using the online tool Tandem Repeats Finder (TRF; Benson, 1999) under default parameters. REPuter (Kurtz et al., 2001) was employed to identify four types of long repeats: forward (F), palindromic (P), reverse (R), and complementary (C) repeats. Minimal repeat size was established at 30, with maximum computed repeats set to 5000, and the hamming distance maintained at three.

RNA editing sites (focusing on cytosine (C) to uracil (U) conversions) within all PCGs of the mitochondrial genome of *H. subcapitata* were predicted via PMGA (Li et al., 2024), and only sites with a probability threshold of ≥ 0.9 were retained for subsequent analyses.

### 2.3 Chloroplast genome assembly and annotation

In this study, the chloroplast genome of *H. subcapitata* was also assembled using OATK v1.0 (Zhou et al., 2025) from the same PacBio HiFi reads from Wang et al. (2025). The assembled genomes were checked and visualized in Bandage v0.7.1 (Wick et al., 2015). Finally, the chloroplast genome was annotated using CPGAVAS2 (Shi et al., 2019) and manually checked in Geneious v2025.0.2 (Kearse et al., 2012) with the published chloroplast genome of *H. subcapitata* (GenBank accession number: PX505294) as a reference.

### 2.4 Homology analysis

BLASTN v2.13.0 (Chen et al., 2015) was employed to align the mitochondria and chloroplast sequences with an e-value threshold of 1e-5 for the identification of mitochondrial plastid DNAs (MTPTs). For the resulting high-scoring pairs, mitogenomic sequences and their flanking regions were extracted as reference sequences. The same protocol was adopted for the identification of nuclear mitochondrial DNAs (NUMTs), and the chromosome-level genome data of *H. subcapitata* was derived from Wang et al. (2025). Finally, the distributions of MTPTs and NUMTs across all genomes were visualized using the Circos package (Zhang et al., 2013) integrated in TBtools v2.309 (Chen et al., 2020).

### 2.5 Analyses of selective pressure and nucleotide diversity

To investigate the selection pressure patterns of mitochondrial PCGs in karstic and non-karstic species, we sampled a total of 27 Lamiales species (including 8 karstic and 19 non-karstic), representing diverse life forms (**Supplementary Table 1**). The karstic group comprised four lithophytic Gesneriaceae species, one holoparasitic Orobanchaceae species, two woody Oleaceae species, and one herbaceous Verbenaceae species. The non-karstic group included one halophytic mangrove species (*Avicennia marina*; Acanthaceae), one carnivorous species (*Genlisea tuberosa*; Lentibulariaceae), one hemiparasitic species (*Castilleja paramensis*; Orobanchaceae), other 10 herbaceous species and six woody species. The PCGs of each mitochondrial genome were obtained using Phylosuite v1.2.2 (Zhang et al., 2020a). The 37 mitochondrial PCGs were aligned separately using MAFFT v7.505 (Katoh and Standley, 2013), and the nonsynonymous/synonymous ratios (Ka/Ks) were determined independently within each group by KaKs_Calculator v2.0 (Wang et al., 2010).

To further detect the hypervariable regions, nucleotide diversity (Pi value) of each PCG was evaluated for the 8 karstic and 19 non-karstic species using DnaSP v6.12.03 (Rozas et al., 2017), respectively. The resulting Pi values of PCGs were visualized as a line graph using R package “ggplot2” (Wickham, 2016).

### 2.6 Investigation of nucleotide substitution spectrum

To identify lineage-specific nucleotide substitutions in PCGs of the 49 sampled Lamiales species (**Supplementary Table 1**), we selected the mitochondrial genome of *Solanum melongena* (GenBank accession number: NC_083979) in Solanales as an outgroup, based on the angiosperm phylogenetic framework placing Solanales as the sister order to Lamiales (Li et al., 2021b). Detailed procedures for identifying and quantifying substitution events followed the methods in Fan et al. (2022). For each position in the concatenated PCG matrix, if the outgroup and a given ingroup species had a different unambiguous base at the same position, a substitution event was recorded. Positions containing gaps or ambiguous bases in either the outgroup or the ingroup sequence were excluded. The absolute frequency of each substitution type was shown by its ratio of the total numbers. The percentage of each substitution type (A↔G, C↔T, A↔C, A↔T, G↔C, G↔T) was calculated by dividing its count by the total number of substitution events recorded for each ecological group. The resulting percentages were visualized using R package “ggplot2” (Wickham, 2016).

### 2.7 Phylogenetic analysis and conflict assessment

To investigate the phylogenetic conflicts of Lamiales based on mitochondrial and chloroplast genomes, mitochondrial and chloroplast genomes of 49 Lamiales species representing 11 families were retrieved from the GenBank (**Supplementary Table 1**). A total of 37 mitochondrial PCGs and 75 chloroplast PCGs shared by these species were extracted using PhyloSuite v1.2.2 (Zhang et al., 2020a) and aligned with MAFFT v7.505 (Katoh and Standley, 2013), respectively. We conducted incongruence length difference (ILD; Farris et al., 1995) test in PAUP v4.b10 (Swofford, 2002) to test the homogeneity within mitochondrial and chloroplast PCGs, respectively. Both *P*-values exceeded 0.01, indicating no significant incongruence between partitions of the two datasets. Therefore, we concatenated the mitochondrial and chloroplast PCGs respectively, and obtained two matrices using Phylosuite v1.2.2 (Zhang et al., 2020a). We selected the best nucleotide substitution model using jModelTest2 (Darriba et al., 2012) under the Akaike information criterion (AIC). Phylogenetic reconstruction was performed using the maximum likelihood (ML) method in RAxML v8.2.12 (Stamatakis, 2014) under the best-fitted GTRGAMMA model. *Solanum melongena* (Solanaceae, Solanales) was selected as the outgroup of Lamiales and the nodal support was assessed based on 1000 bootstrap (BS) replicates.

Subsequently, individual mitochondrial gene trees were also constructed using RAxML v8.2.12 (Stamatakis, 2014) under the same settings. Owing to the absence of six mitochondrial PCGs in the outgroup, only 31 PCGs yielded reliable single gene trees, which were integrated to infer the species tree using ASTRAL v4 (Zhang et al., 2018). Similarly, *S. melongena* was designated as outgroup for rooting, and the -u 2 option was enabled to output normalized quartet concordance scores. To quantify the contributions of ILS and introgression/hybridization (IH) among these Lamiales species, PhyTop v0.3 (Shang et al., 2025) was employed based on the obtained species tree with default parameters. We calculated the ILS and IH indices using PhyTop based on the proportions of the three possible quartet topologies (q1, q2, q3) for each node, with higher ILS-e index or IH-e index values indicating stronger signals for the respective process.

## 3 Results

### 3.1 Features of the mitochondrial genome of *H. subcapitata*

**Figure 1:**
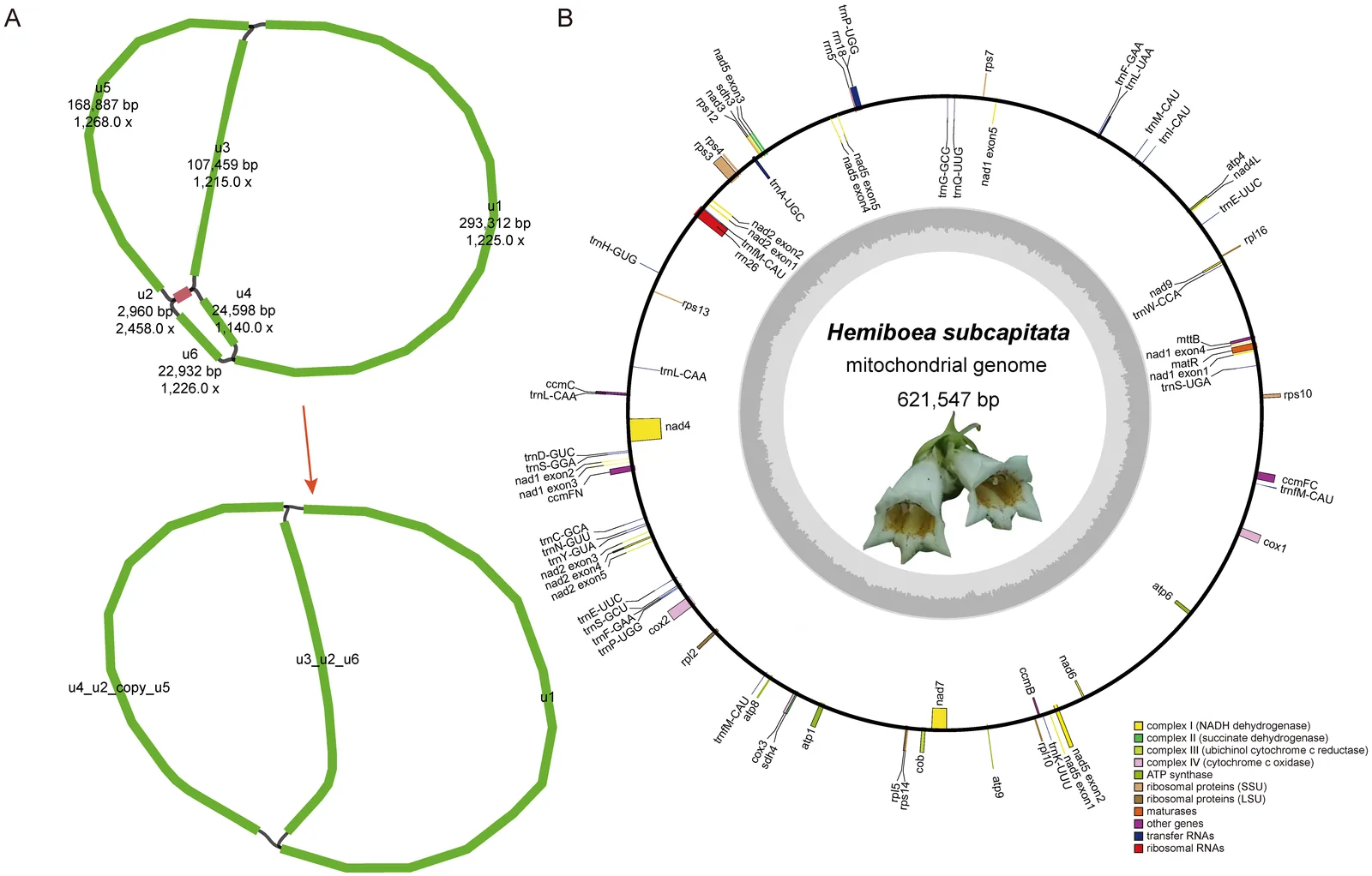
Assembly and annotation of H. subcapitata mitochondrial genome. **(A)** Schematic of *H. subcapitata* mitochondrial genome assembly. Contig lengths and sequencing coverage are labelled for each segment. The adjacency relationships among contigs were supported by long-read sequencing data, and a pair of alternative genomic conformations generated by repeat-mediated homologous recombination were plotted. **(B)** The mitochondrial genome map of *H. subcapitata* with annotation. Genes with different functions are depicted using different colors.

*De novo* assembly of the PacBio HiFi reads was preliminarily generated into six primary unitigs, which were subsequently integrated into an assembly graph based on long-read overlapping signals (**Figure 1A**). All adjacency connections between unitigs are supported by continuous spanning long reads. Read mapping against the assembled genome revealed a mean sequencing depth of 1887.22 × (ranging from approximate 1140× to 2458× across the six unitigs), supporting the high quality and reliability of the assembly. The final assembly yielded the complete mitochondrial genome structure of *H. subcapitata* (**Figure 1A**), which is a simple circular molecule of 621,547 bp with a 43.6% GC content (**Figure 1B**). A total of 67 genes were annotated, comprising 37 PCGs, 3 rRNAs, and 27 tRNAs (**Table 1**). Within the 37 genes, eight genes were related to the cytochrome c system, including one *cob* gene, three *cox* genes encoding cytochrome c oxidase subunits, and four *ccm* genes responsible for cytochrome c biogenesis. Eleven genes to dehydrogenase activity, including nine *nad* genes encoding NADH dehydrogenase subunits and two *sdh* genes encoding succinate dehydrogenase subunits. Five genes to ATP synthase, 11 genes to ribosomal proteins (four *rpl* genes and seven *rps* genes), and two genes to maturation and protein transport (*matR* and *mttB*).

**Table 1:** Gene contents of the mitochondrial genome of *H. subcapitata*.

Group of genes	Name of genes
ATP synthase	*atp1*, *atp4*, *atp6*, *atp8*, *atp9*
NADH dehydrogenase	*nad1*, *nad2*, *nad3*, *nad4*, *nad4L*, *nad5*, *nad6*, *nad7*, *nad9*
Cytochrome b	*cob*
Cytochrome c biogenesis	*ccmB*, *ccmC*, *ccmFC*, *ccmFN*
Cytochrome c oxidase	*cox1*, *cox2*, *cox3*
Maturases	*matR*
Protein transport subunit	*mttB*
Ribosomal protein large subunit	*rpl2*, *rpl5*, *rpl10*, *rpl16*
Ribosomal protein small subunit	*rps3*, *rps4*, *rps7*, *rps10*, *rps12*, *rps13*, *rps14*
Succinate dehydrogenase	*sdh3*, *sdh4*
Ribosome RNA	*rrn5*, *rrn18*, *rrn26*
Transfer RNA	*trnA^UGC^*, *trnC^GCA^*, *trnD^GUC^*, *trnE^UUC^* (× 2), *trnF^GAA^* (× 2), *trnfM^CAU^* (×3), *trnG^GCC^*, *trnH^GUG^*, *trnI^CAU^*, *trnK^UUU^*, *trnL^CAA^* (× 2), *trnL^UAA^*, *trnM^CAU^*, *trnN^GUU^*, *trnP^UGG^*(× 2), *trnQ^UUG^*, *trnS^GGA^*, *trnS^GCU^*, *trnS^UGA^*, *trnW^CCA^*, *trnY^GUA^*

**Figure 2:**
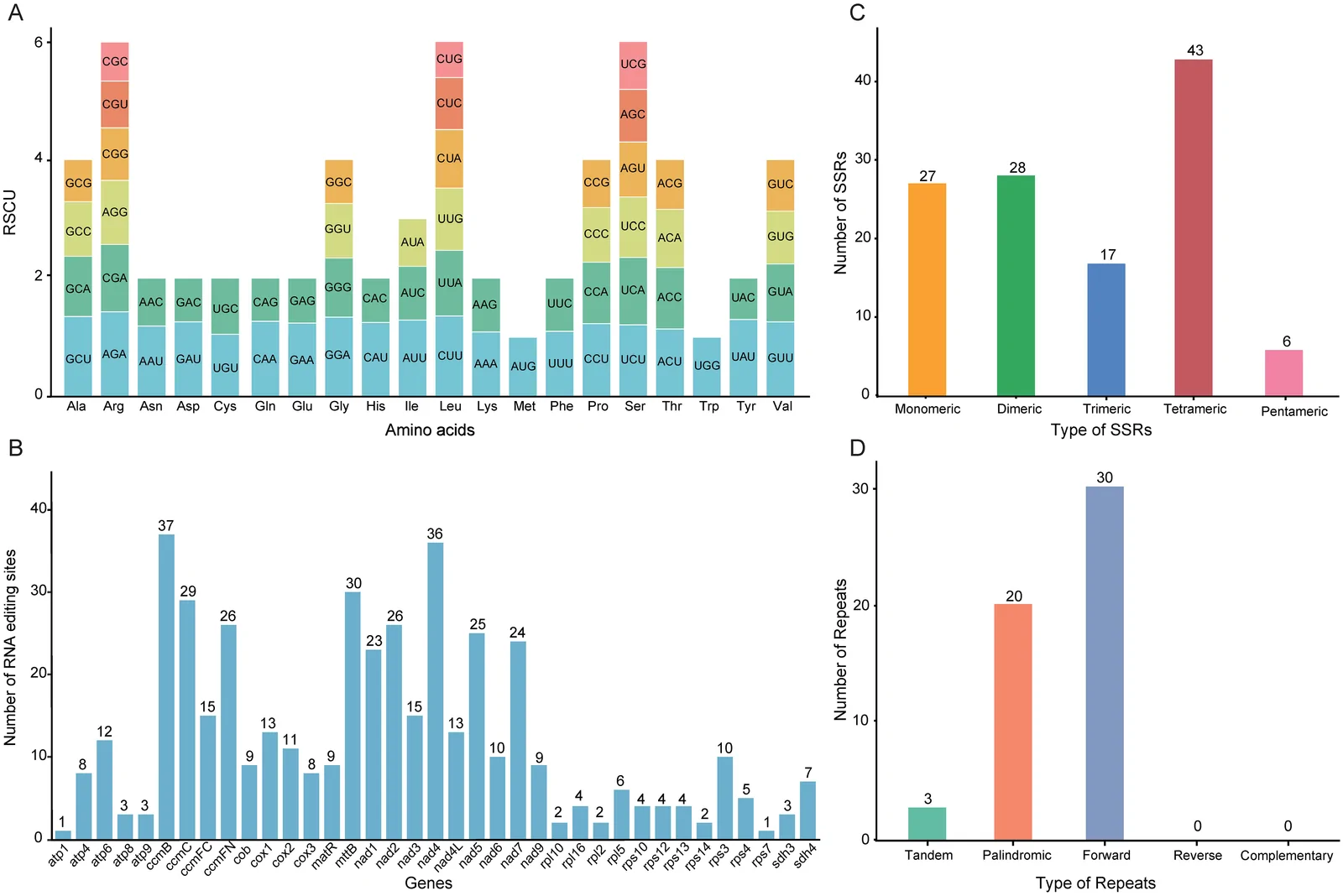
Genomic features of the *H. subcapitata* mitochondrial genome. **(A)** RSCU values of amino acids in the mitochondrial PCGs. **(B)** Number of RNA editing sites identified in each PCGs. **(C)** SSR types and the number of repetitive fragments identified in mitochondrial genome. **(D)** Repeat types and the count of repetitive fragments identified in the mitochondrial genome.

We detected 27 high-frequency codons with RSCU > 1, 31 codons with RSCU < 1, and three codons (AUG, UGG, and GGG) exhibited RSCU = 1 (**Figure 2A**, **Supplementary Table 2**). Among them, AGA (Arg) exhibited the highest RSCU value at 1.43, while CUG (Leu) showed the lowest at 0.61 (**Supplementary Table 2**).

A total of 450 C-to-U RNA editing sites were detected within the 37 PCGs of the *H. subcapitata* mitogenome (**Figure 2B**). Among them, *ccmB* possessed the greatest number of editing sites (37 sites), followed by *nad4* with 36 sites, whereas only one editing site was observed in *atp1* and *rps7* (**Supplementary Table 3**). Among all editing events, hydrophilic-to-hydrophobic conversions accounted for the largest proportion (46.67%, 210 sites), followed by hydrophobic-to-hydrophobic changes (30.00%, 135 sites) and hydrophilic-to-hydrophilic substitutions (13.56%, 61 sites). Hydrophobic-to-hydrophilic conversions represented 9.33% (42 sites) of the total changes and 0.44% (2 sites) of RNA editing events led to the generation of stop codons (**Supplementary Table 4**).

A total of 121 SSRs were identified in the mitochondrial genome of *H. subcapitata* (**Supplementary Table 5**). Tetrameric repeats were the most abundant (43, 35.53%), followed by dimeric (28, 23.14%), monomeric (27, 22.31%), trimeric (17, 14.05%), and pentameric repeats (6, 4.96%) (**Figure 2C**). Additionally, three tandem repeats (**Supplementary Table 6**), 30 forward repeats and 20 palindromic repeats (**Supplementary Table 7**) were identified in the *H. subcapitata* mitogenome (**Figure 2D**).

### 3.2 Numbers of mitochondrial-plastid DNAs and mitochondrial-nuclear DNAs

**Figure 3:**
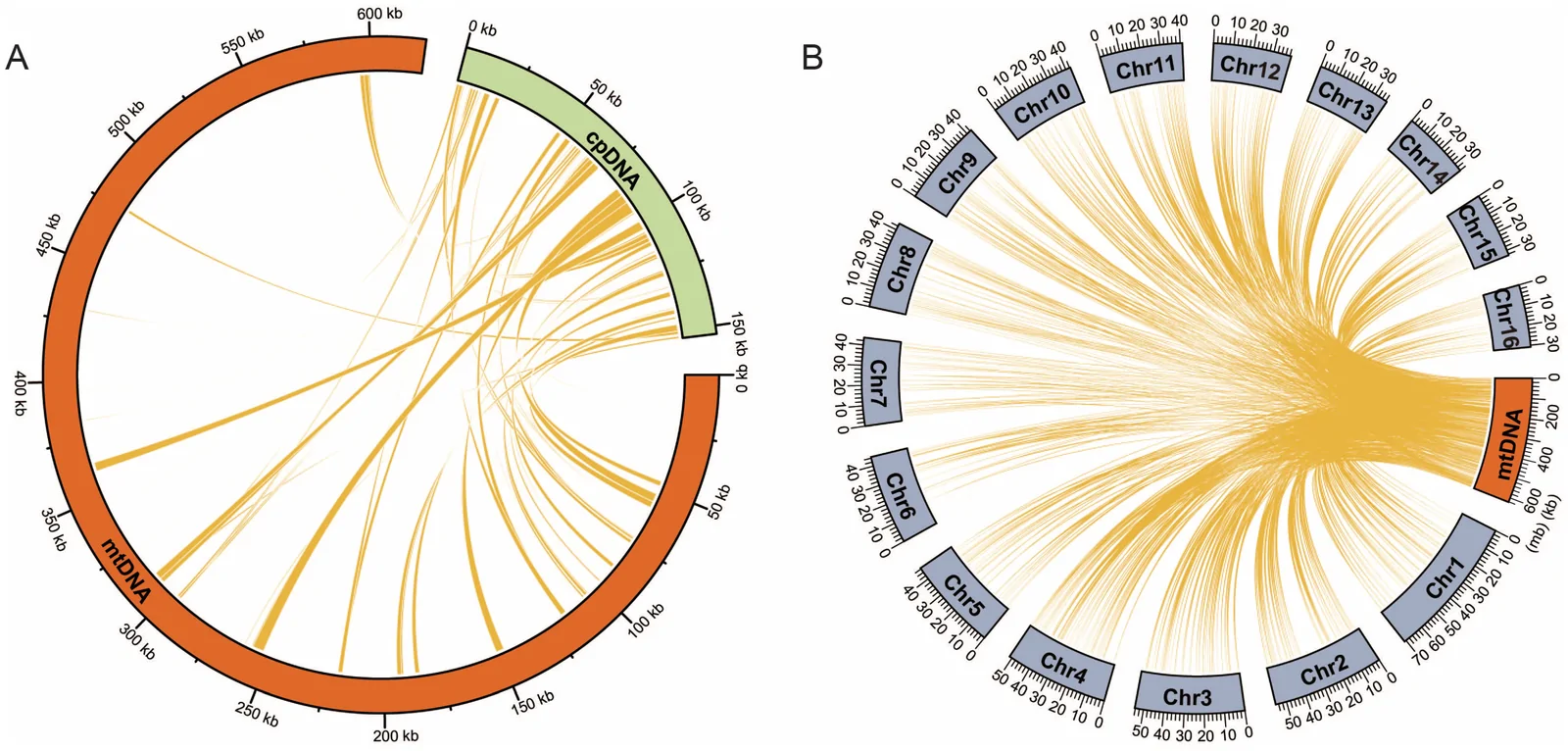
Shared homologous sequences between the mitochondrial genome and other genomes in *H. subcapitata.* **(A)** Similar sequences shared between the mitochondrial genome (mtDNA) and chloroplast genome (cpDNA). The orange and green arcs represent the mtDNA and cpDNA, respectively. The inner circle arcs represent MTPTs. **(B)** Similar sequences shared between the mtDNA and sixteen chromosomes genome (Chr1-Chr16) of the nuclear genome. The orange arcs and grey arcs represent the mtDNA and nuclear chromosomal sequences. The inner circle arcs represent NUMTs.

The new assembled chloroplast genome of *H. subcapitata* exhibited a length of 153,193 bp. A total of 75 mitochondrial-plastid DNAs (MTPTs, 72,737 bp) were identified in the mitochondrial genome of *H. subcapitata*, accounting for 11.70% of the mitogenome length (**Figure 3A**). Based on compositional complexity, 75 MTPTs were classified into two major categories. The non-chimeric group comprised 59 MTPTs, including 3 complete PCGs, 34 partial PCGs, 6 complete tRNAs, 11 intergenic spacer (IGS) regions, and 5 partial rRNA fragments. The chimeric group comprised 16 MTPTs: 7 MTPTs with both complete and partial PCGs, 3 MTPTs with complete PCGs and complete tRNAs, 3 MTPTs with partial tRNAs and partial PCGs, 1 MTPTs with complete PCGs together with partial tRNAs and partial PCGs, 1 MTPTs with complete tRNAs along with partial tRNAs and complete PCGs, and 1 MTPTs with complete tRNAs and partial rRNA (**Supplementary Table 8**).

In addition, a total of 935 mitochondrial-nuclear DNAs (NUMTs) were identified in the nuclear genome of *H. subcapitata*, with lengths ranging from 35 bp to 6852 bp (**Figure 3B**). Specifically, 154 NUMTs were from Chr4, followed by Chr15 (78), Chr1 (77), Chr3 (73) and Chr2 (71). Among them, 930 were non-chimeric NUMTs, including 38 complete PCGs, 2 complete tRNAs and 828 IGS regions, 32 partial PCGs and 30 partial tRNA fragments; the remaining 5 NUMTs were chimeric, including 4 fragments with both partial rRNAs and partial PCGs, one fragment with both partial tRNAs and partial rRNA (**Supplementary Table 8**).

### 3.3 Comparison of mitochondrial genomes between karstic and non-karstic species in Lamiales

**Figure 4:**
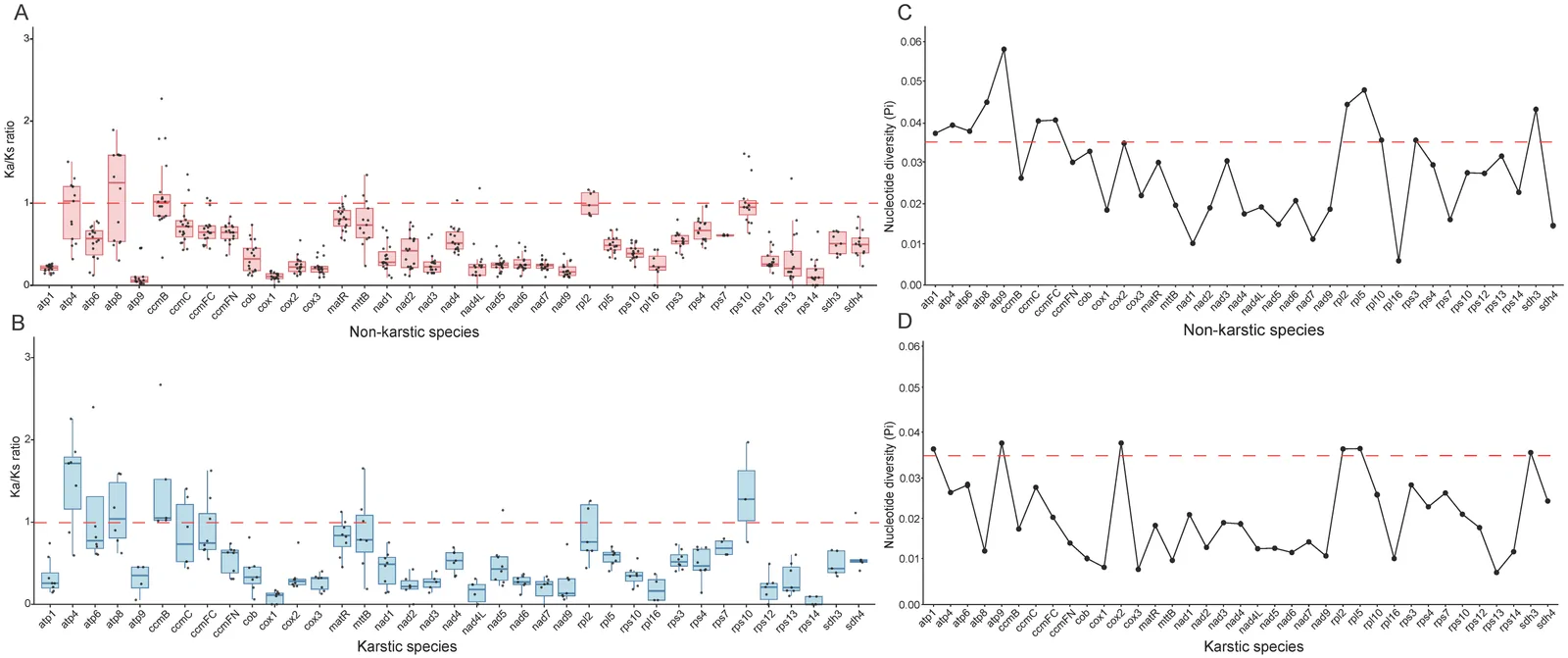
Comparison of selective pressure and nucleotide diversity for mitochondrial PCGs between karstic and non-karstic species. **(A)** Boxplots of Ka/Ks ratios in non-karstic species. **(B)** Boxplots of Ka/Ks ratios for mitochondrial PCGs in karstic species. The horizontal red dashed line marks the evolutionary threshold (Ka/Ks = 1); **(C)** Line plots of Pi values for each mitochondrial PCG in non-karstic species. **(D)** Line plots of Pi values for each mitochondrial PCG in karstic species. Red dashed line is set at Pi = 0.035.

Among the 37 mitochondrial PCGs, the Ka/Ks ratios of three genes (*atp4*, *atp8* and *ccmB*) were greater than 1, and the remaining were less than 1 in non-karstic species (**Figure 4A**). For karstic species, the Ka/Ks ratios of 33 mitochondrial PCGs were less than 1, and the ratios of *atp4*, *atp8*, *ccmB* and *rps10* were greater than 1 (**Figure 4B**; **Supplementary Table 9**).

The Pi values of non-karstic species ranged from 0.00599 (*rpl16*) to 0.05817 (*atp9*) (**Supplementary Table 10**). Twelve highly variable PCGs (Pi > 0.035) were detected, i.e., *atp9* (0.05817), *rpl5* (0.04812), *atp8* (0.04509), *rpl2* (0.04454), *sdh3* (0.04334), *ccmFC* (0.04069), *ccmC* (0.04047), *atp4* (0.03943), *atp6* (0.03794), *atp1* (0.03741), *rpl10* (0.03574), *rps3* (0.03571) (**Figure 4C**). For karstic species, Pi values spanned from 0.00743 (*rps13*) to 0.03747 (*cox2*) (**Supplementary Table 10**), and six highly variable PCGs (Pi > 0.035) were identified, i.e., *cox2* (0.03747), *atp9* (0.03746), *rpl15* (0.03618), *rpl12* (0.03609), *atp1* (0.03609) and *sdh3* (0.03526) (**Figure 4D**). Notably, five PCGs (*atp1, atp9, rpl2, rpl5* and *sdh3*) exhibited high Pi values in both karstic and non-karstic species.

**Figure 5:**
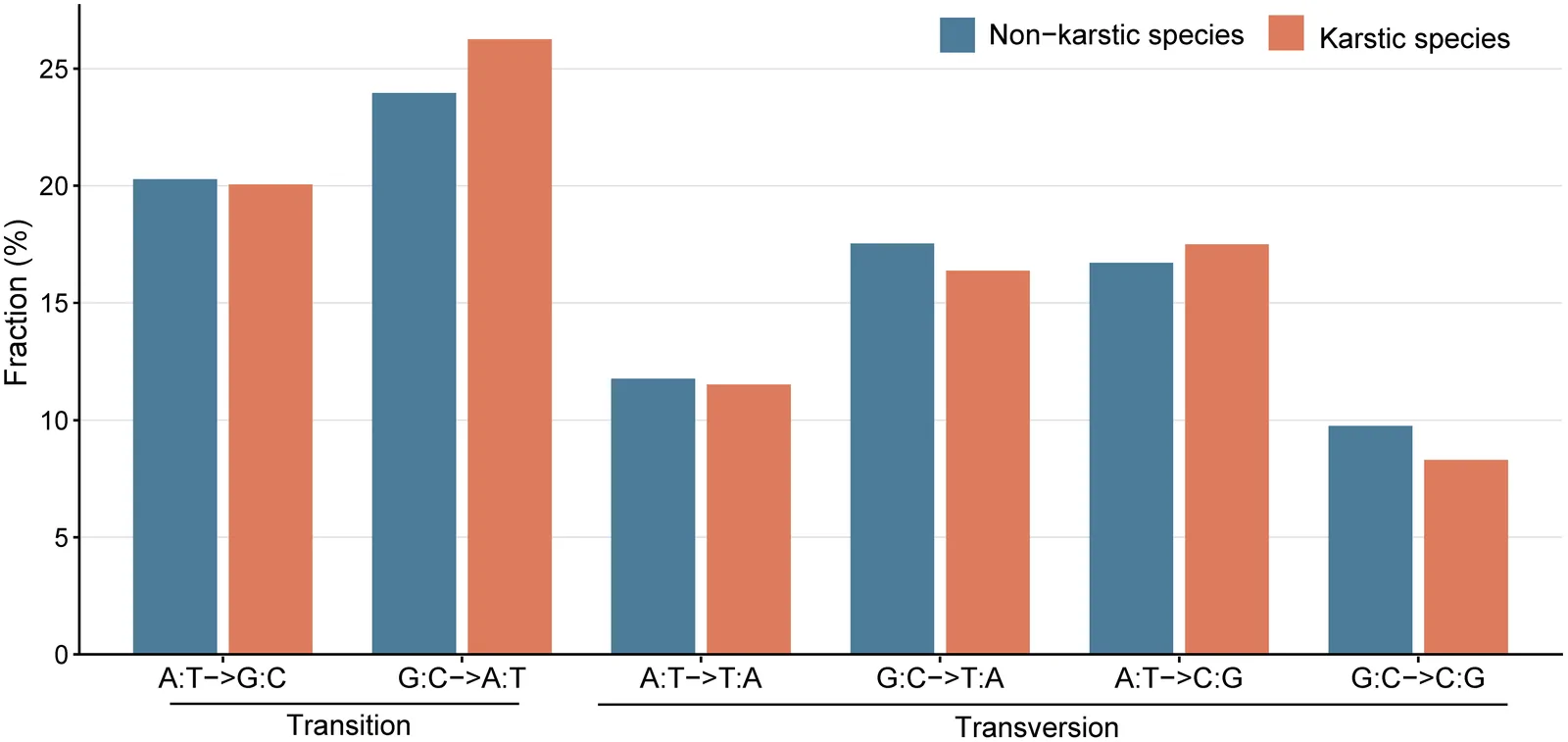
Mutation spectra in the mitochondrial genome of karstic and non-karstic Lamiales species. The y-axis represents the fraction (%) of each type of nucleotide substitution. Substitutions are classified into transitions (A:T→G:C, G:C→A:T) and transversions (A:T→T:A, G:C→T:A, A:T→C:G, G:C→C:G) as labelled on the x-axis.

Regarding the mutational spectrum, G:C→A:T transition (23.96%) and A:T→G:C transition (20.28%) were the two most frequent substitution types for non-karstic species. G:C→T:A (17.53%) transversion was slightly more frequent than A:T→C:G (16.71%), while A:T→T:A (11.77%) and G:C→C:G (9.75%) transversions had the lowest proportions (**Figure 5**). For karstic species, the most frequent base substitution was also G:C→A:T transition (26.26%), followed by A:T→G:C transition (20.05%). Among all transversions, A:T→C:G (17.50%) and G:C→T:A (16.37%) were more frequent than A:T→T:A (11.52%) and G:C→C:G (8.30%) (**Supplementary Table 11**).

### 3.4 Phylogeny and chloroplast-mitochondrial conflicts within Lamiales

**Figure 6:**
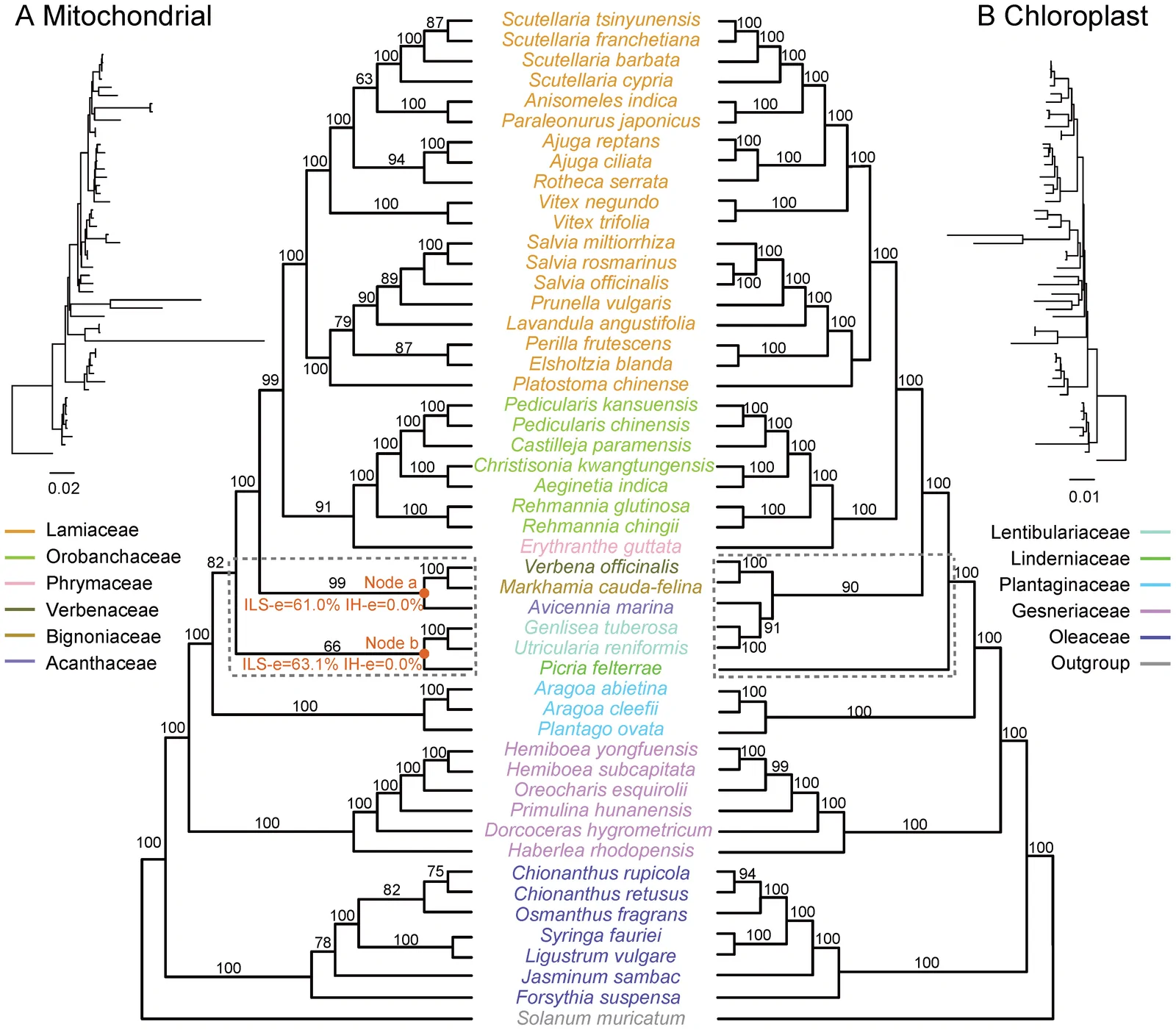
Phylogenetic relationships of Lamiales species inferred from mitochondrial genome **(A)** and chloroplast genome **(B)**. Numbers above the branches are ML-BS values. The nodes with cytonuclear discordance were marked in red. ILS-e (estimated incomplete lineage sorting index) and IH-e (estimated introgression/hybridization index) represent the proportion of gene tree topological discordance that can be explained by the incomplete lineage sorting **(ILS)** and hybridization, respectively.

The 37 mitochondrial PCGs dataset displayed 57,105 characters, 7928 of which were parsimony informative. The 75 chloroplast PCGs dataset had 60,675 characters, 8628 of which were parsimony informative. The topologies based on 37 mitochondrial PCGs and 75 chloroplast PCGs were basically concordant. Each sampled family of Lamiales was a well-supported monophyletic with strongly supporting values (**Figure 6**). Further, the phylogenetic relationships among families were resolved well, however, two discordances were identified within Verbenaceae, Bignoniaceae, Acanthaceae, Lentibulariaceae and Linderniaceae (**Figure 6**). Such as Acanthaceae was sister to a clade including Verbenaceae and Bignoniaceae (ML-BS = 99%) based on 37 mitochondrial PCGs, while Acanthaceae and Lentibulariaceae formed a clade (ML-BS = 91%) (**Figure 6**, node a). Furthermore, the contributed values of ILS events (ILS-e) were 61.0% and 63.1%, respectively.

## 4 Discussion

### 4.1 Mitochondrial genome feature of *H. subcapitata*

In this study, we newly reported the mitochondrial genome of *H. subcapitata* (Gesneriaceae), with a size of 621,547 bp and a GC content of 43.6% (**Figure 1B**). There are 67 genes annotated in mitochondrial genomes of *H. subcapitata*, including 37 PCGs, 3 rRNA genes, and 27 tRNA genes. Among five Gesneriaceae species reported in previous studies (Zhang et al., 2012; Baev et al., 2021; Chen et al., 2024, Chen et al., 2025; Tao et al., 2026), the mitochondrial genome lengths varied from 454,871 bp in *Oreocharis esquirolii* (Chen et al., 2025) to 619,997 bp in *H. yongfuensis* (Tao et al., 2026). Additionally, the GC content of the mitochondrial genomes in Gesneriaceae ranged from 43.3% (*Dorcoceras hygrometricum*) to 44.1% (*Haberlea rhodopensis*). The total annotated gene numbers ranged from 59 in *Hab. rhodopensis* to 68 in *Primulina hunanensis*. Specifically, the numbers of PCGs varied from 32 in *D. hygrometricum* to 37 in *H. subcapitata* and *H. yongfuensis* (**Supplementary Table 12**). Overall, the gene size and gene numbers of the mitochondrial genome were diverse, while the GC content was conserved in Gesneriaceae mitochondrial genomes.

The mitochondrial genome of *H. subcapitata* was found to be a typical circular genome structure (**Figure 1**). Among the published Gesneriaceae mitochondrial genomes, both linear conformations (*H. yongfuensis*, *P. hunanensis*, and *O. esquirolii*) and circular conformations (*D. hygrometrica* and *Hab. rhodopensis*) have been observed. Though *H. subcapitata* is closely related to *H. yongfuensis*, the mitochondrial genomes of them displayed different structures, which indicated high complexity of mitochondrial genome structures in Gesneriaceae.

Codon usage bias is an important factor influencing translation efficiency and gene expression (Parvathy et al., 2022). Among the 27 codons with RSCU > 1, the most preferred codon was AGA (Arg, RSCU = 1.43), followed by CUU (Leu, RSCU = 1.37) and GCU (Ala, RSCU = 1.35) (**Figure 2A**). That is consistent with a preference for A/U-ending codons in mitochondrial genomes of angiosperms (Sloan and Wu, 2014). Such preference.is thought to reflect selection for efficient and accurate translation (Majeed et al., 2020; Plotkin and Kudla, 2011).

In *H. subcapitata*, 450 RNA editing sites were detected across 37 mitochondrial PCGs (**Figure 2B**), which is similar with the results of other Gesneriaceae species, such as *P. hunanensis* (455 RNA editing sites; Chen et al., 2024) and *H. yongfuensis* (429 RNA editing sites; Tao et al., 2026). The highest density editing genes were *ccmB* (37 sites) and *nad*4 (36 sites). In addition, editing events that generating premature stop codons were identified in *atp6* and *rps10*. Comparing with the closely related species *P. hunanensis* (Chen et al., 2024) and *H. yongfuensis* (Tao et al., 2026), we found that the majority of these sites (more than 90%) are conserved at homologous positions. Moreover, the evolutionary conservation of these sites, which is related to driving hydrophobicity remodeling and premature stop codon generation, might exert regulatory effects on mitochondrial gene expression.

In the mitochondrial genome of *H. subcapitata*, tetrameric SSRs and forward repeats were the predominant repeats types (**Figures 2C, D**), which was consistent with previous reports in other Lamiales lineages (e.g., *Cistanche*, Miao et al., 2022; *P. hunanensis*, Chen et al., 2024). Among the identified dispersed repeats, the length of the longest one was 4556 bp (**Supplementary Table 7**), which implied that the presence of long repetitive elements may contribute to elevated sequencing depth in repeat-rich regions. Notably, the sequencing depth across the six primary unitigs varied considerably, ranging from approximately 1140× to 2458×, which might reflect the uneven distribution of long repeats among these unitigs. The similar phenomenon has been observed in *Corydalis ophiocarpa* (Papaveraceae) (Lei et al., 2026), which found that a 6383 bp repeat was associated with elevated sequencing depth in the complete mitochondrial genome.

We identified 75 MTPTs in the mitogenome of *H. subcapitata* (**Figure 3A**), and these MTPTs were from PCGs, tRNA genes, and IGS of the chloroplast genome (**Supplementary Table 6**). The coexistence of intact and fragmented genes within the same mitogenome is common and has been widely documented across angiosperms (Alverson et al., 2010; Yurina and Odintsova, 2016; Nhat Nam et al., 2024). Furthermore, there were 935 NUMTs detected, and these NUMTs distributed across all 16 chromosomes (Chr1-Chr16) with high density on Chr4 (**Figure 3B**, **Supplementary Table 6**). Preferential distribution may be associated with chromatin state, recombination rate, and the abundance of repetitive sequences, and these three factors collectively shaped the non-random integration of organellar DNA into the nuclear genome (Zhang et al., 2020b). Similar uneven distribution patterns of NUMTs have been documented in diverse plant species (e.g., Richly and Leister, 2004; Michalovova et al., 2013).

### 4.2 Comparison of mitochondrial genomes of karstic and non-karstic species

The Ka/Ks ratio is always employed to assess the selective pressure acting on PCGs, which determines the effects of environmental stress on the evolution of the mitochondrial genome (Qiao et al., 2022). Among the 37 mitochondrial PCGs, 90% of them had the Ka/Ks ratios less than 1. Nevertheless, the Ka/Ks ratios of three PCGs (*atp4*, *atp8* and *ccmB*) and four PCGs (*atp4*, *atp8*, *ccmB* and *rps10*) were more than 1 in non-karstic and karstic species, respectively. The Ka/Ks values of *rps10* in all karstic species with diverse life forms were greater than 1, which might indicate that this gene has undergone positive selection. The ribosomal protein gene *rps10* encodes the S10 protein, a core component of the small subunit of mitochondrial ribosomes that is essential for organellar translation (Kwasniak et al., 2013). Previous research found that *rps10* in mitochondrial genome of *O. esquirolii* is potentially related to its adaptation to environmental stressors (Chen et al., 2025). Similarly, the Ka/Ks value of *rps10* was greater than 1 in the desert legume *Sophora alopecuroides*, implying its potential role in adaptation to drought and salinity stress (Li et al., 2025). Therefore, we speculated that *rps10* exhibited elevated Ka/Ks ratios exclusively in karstic species, which might be related with the unique environmental challenges of karst habitats.

Furthermore, our results revealed that Pi values of 12 PCGs of non-karstic species were more than 0.035, while six PCGs were more than 0.035 in karstic species. Of these, five PCGs (*atp1, atp9*, *rpl2, rpl5* and *sdh3*) had high Pi values in both karstic and non-karstic species, which could be used as candidate molecular markers for species identification of Lamiales. The mutational spectrum revealed that transitions accounted for 53.69% and 55.76% of nucleotide substitutions in karstic and non-karstic Lamiales mitochondrial genomes, respectively. G:C→A:T transition was the most frequent single substitution type. Among all transversions, A:T→C:G transversion was slightly more frequent than G:C→T:A transversion, with G:C→C:G transversion being the rarest. Notably, the mutational spectra of karstic and non-karstic species were remarkably similar, which suggests that habitat-specific selective pressures in karstic regions—including high soil calcium availability, frequent periodic drought, and chronic oxidative stress—have not substantially altered the fundamental mutation spectrum of plant mitochondrial genomes.

### 4.3 Phylogenetic conflicts between organellar genomes in Lamiales

Our phylogenetic results based on mitochondrial genomes and chloroplast genomes consistently supported the monophyly of Lamiales (**Figure 6**). The relationships between families derived from mitochondrial genomes and chloroplast genomes were basically consistent with previous studies based on same molecular data (Li et al., 2021b; Tao et al., 2026). Although both plastids and mitochondria were maternally inherited, an increasing number of incongruent topologies were detected between mitochondrial and chloroplast data in various taxonomic ranks of angiosperms, such as Rosidae (Sun et al., 2015), Pandanales (Soto Gomez et al., 2020), Rubiaceae (Rydin et al., 2017), *Potentilla* (Rosaceae; Xue et al., 2024). Notably, we detected phylogenetic conflict between the phylogenies derives from these two organellar genomes in Verbenaceae, Bignoniaceae, Acanthaceae, Lentibulariaceae and Linderniaceae (Nodes a and b in **Figure 6**). Similarly, phylogenetic conflicts between 353 nuclear genes and chloroplast genomes were also detected in Verbenaceae, Bignoniaceae, Acanthaceae, Lentibulariaceae (Li et al., 2021b; Zuntini et al., 2024). In addition, we found that ILS might be a major contributor to the observed phylogenetic discordance between the two organellar genomes (**Figure 6**). Indeed, ILS was also considered as the main contributor of the cytonuclear conflict in Lamiales, such as Bignoniaceae (Dong et al., 2022; Yan et al., 2026) and Gesneriaceae (Yang et al., 2023). Further evidence is needed to deeply investigate the reasons behind ILS and phylogenetic conflicts between organellar genomes in Lamiales.

## 5 Conclusion

We assembled and characterized the complete mitochondrial genome of *H. subcapitata*, a circular molecule of 621,547 bp with 67 genes. Comparative analyses revealed substantial genome size variation and gene composition but conserved GC content among Gesneriaceae mitochondrial genomes. In the pattern of selective pressure, three genes (*atp9*, *atp1* and *sdh3*) exhibited elevated Ka/Ks ratios across Lamiales, and the Ka/Ks value of *rps10* was above 1 only in karstic species. We selected five highly variable genes (Pi > 0.035) for candidate molecular markers. Phylogenomic analyses uncovered discordance between mitochondrial and chloroplast trees, which was predominantly related with ILS. Collectively, these findings would be helpful to understand the evolutionary changes of mitochondrial genome in karstic plants. Besides, the limited taxonomic sampling should be further strengthened in future studies to validate the generality of the comparative conclusions.

## Data Availability

The mitochondrial and chloroplast genomes reported in this study have been deposited in China National Center for Bioinformation (CNCB; https://ngdc.cncb.ac.cn/gwh/) under accession numbers: CAA452134 and CAA452135.
