# Supplementary material for: The complete mitochondrial genome of Hemiboea subcapitata (Gesneriaceae) and comparative analysis across Lamiales
Source: Front Plant Sci. 2026 Aug 12;17:1921030. doi: 10.3389/fpls.2026.1921030 (PMC13506962; doi:10.3389/fpls.2026.1921030)
Supplement: Supplementary file 1 [file Table1.doc]

**Supplementary Information**

**Table S1.** Accession numbers of mitochondrial genome used in in this study.

**Table S2.** Optimal codons in mitochondrial PCGs of *H. subcapitata*.

**Table S3.** Potential RNA editing sites in mitochondrial PCGs of *H. subcapitata*.

**Table S4.** Amino acid changes in potential RNA editing sites in mitochondrial PCGs of *H. subcapitata*.

**Table S5.** SSRs in the mitochondrial genome of *H. subcapitata.*

**Table S6.** Tandem repeat sequences in mitochondrial genome of *H. subcapitata.*

**Table S7.** Dispersed repeat sequences in the mitochondrial genome of *H. subcapitata*

**Table S8.** The homologous DNA fragment in mitochondrial genome of *H. subcapitata*.

**Table S9.** Ka/Ks values for mitochondrial PCGs in karstic and no-karstic Lamiales species

**Table S10.** Nucleotide diversity (Pi) of mitochondrial PCGs in karstic and non-karstic Lamiales species.

**Table S11.** Nucleotide substitution spectrum of mitochondrial PCGs in karstic and non-karstic Lamiales species

**Table S12.** General features of mitochondrial genomes of six Gesneriaceae species.
